# Role of Overweight and Obesity in Gastrointestinal Disease

**DOI:** 10.3390/nu12010111

**Published:** 2019-12-31

**Authors:** Sara Emerenziani, Michele Pier Luca Guarino, Laura Maria Trillo Asensio, Annamaria Altomare, Mentore Ribolsi, Paola Balestrieri, Michele Cicala

**Affiliations:** Gastroenterology Unit, University Campus Bio-Medico di Roma, via Álvaro del Portillo 21, 00128 Rome, Italy; m.guarino@unicampus.it (M.P.L.G.); a.altomare@unicampus.it (A.A.); m.ribolsi@unicampus.it (M.R.); p.balestrieri@unicampus.it (P.B.); m.cicala@unicampus.it (M.C.)

**Keywords:** overweight and obesity, functional gastrointestinal disorders (FGIDs), visceral hypersensitivity, gastrointestinal motility, cancer

## Abstract

The prevalence of obesity is increasing worldwide, leading to a severe impairment of overall health. Actually, obesity has been associated with several pathological conditions, causing an excess overall mortality. In particular, overweight and obesity are well known risk factors for a variety of gastrointestinal (GI) disorders i.e., functional GI disorders as well as, inflammatory bowel disease (IBD), pancreatitis, and GI cancer. The aim of the present review is to summarize the potential role of overweight and obesity in GI disease with particular focus on plausible biological mechanisms that could explain the association between obesity and GI disease based on the most recent evidence in the literature.

## 1. Introduction

Prevalence of obesity is increasing worldwide and it is becoming one of the major healthissuein adult population, as well as among children and adolescents. The World Health Organization (WHO) defines obesity as excessive body fat accumulation, which is associated with several risks to health. To overcome the difficulties associated with measuring and classifying the percentage of body fat, the WHO established the body mass index (BMI) as the specific parameter to define overweight and obesity. According to BMI, individuals are allocated to overweight or obesity state as it is shown in Table 1.

Nevertheless, BMI does not allow a complete assessment of body composition, as it does not discriminate fat-free mass from adipose tissue. Thus, a subject with normal BMI (18.5–24.9 kg/m^2^) could have either an appropriate body fat percentage or an excess of fat that might be hidden by normal values of BMI [1]. Obesity is associated with a large decrease in life expectancy. It is well known that obesity is a worldwide health concern that leads to an increased risk for several diseases apart from GI disease, most notably cardiovascular diseases, diabetes, and cancers; moreover, in the last years several evidencesuggesta strong association between obesity and a large decrease in life expectancy [2,3,4,5]. Different mechanisms may link obesity and GI disease ranging from mechanical, pro-tumoral, pro-cancerogenic, and dietary factors as shown in Table 2. 

**Figure 1:**
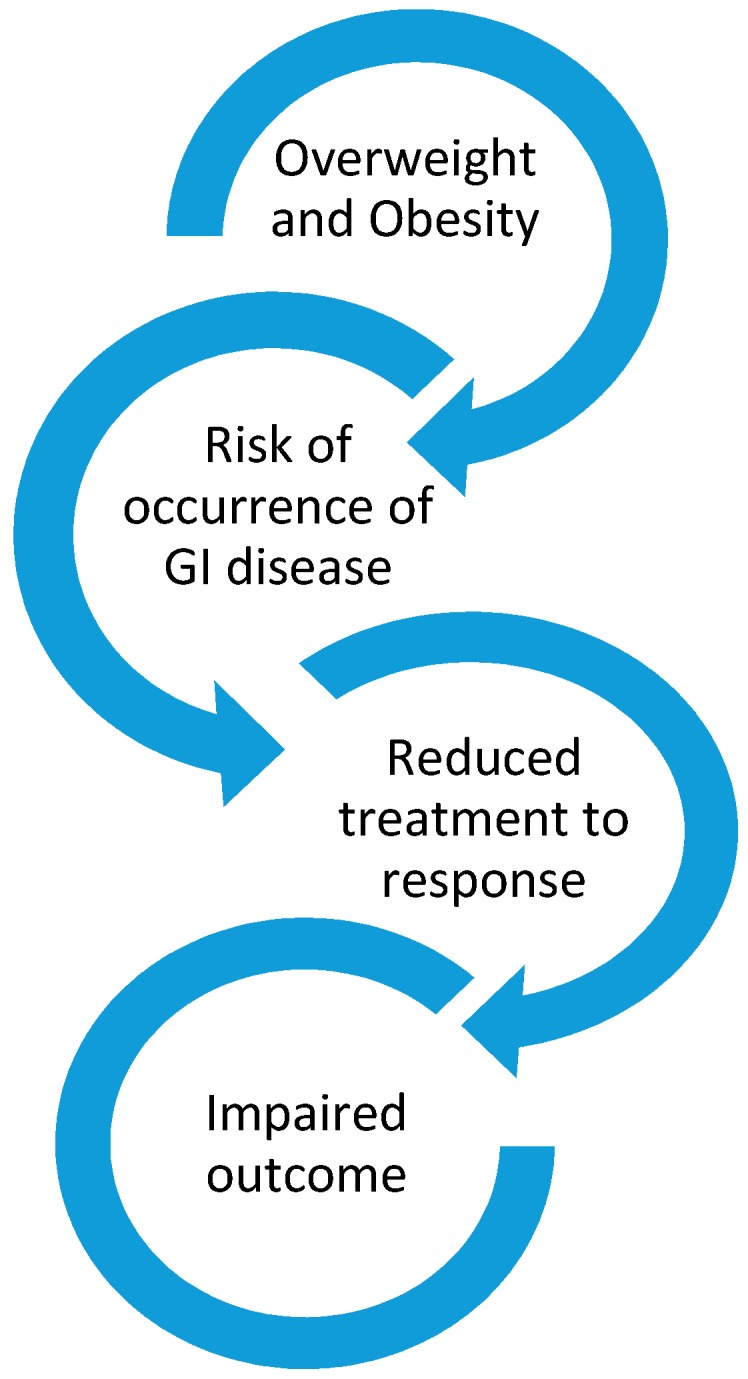
Relationship between obesity occurrence of gastrointestinal (GI) disease and impaired clinical outcome.

Obesity is not only a risk factor for the occurrence of several GI disorders but also can negatively impact clinical outcomes mostly by reducing response to specific treatment. Figure 1.

In general, it has been demonstrated that obesity might affect gastric GI motility. A very recent study has pointed out that peptides secreted by adipocytes, namely, leptin, adiponectin, nesfatin-1, and apelin, are able to modulate GI motility, acting both centrally and peripherally. It is time to emphasize the interplay between the adipose tissue, the central nervous system, and GI tract both in physiological or pathophysiological conditions. Since consistent data from literature support the role of peripheral signals in determining the GI motor responses and the regulation of food intake, this pathway could represent an opportunity to reveal novel therapeutic approaches either for obesity or for GI disease [18]. 

**Figure 2:**
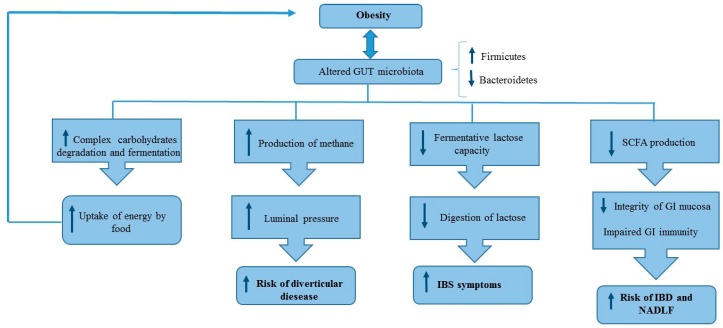
Relationship between altered microbiota composition and different pathophysiological mechanisms of GI disease in obese patients. SCFA: Short chain fatty acids, IBS: Irritable bowel syndrome, IBD: Inflammatory bowel disorders, NAFLD: Non-alcoholic fatty liver disease.

Moreover, both quantitative and qualitative modifications of gut microbiota observed in obese patients are related to several pathophysiological mechanisms, which could explain the relationship between obesity and GI disease as shown in Figure 2.

The aim of the present review is to outline the major plausible mechanisms that could explain the association between overweight/obesity and GI disease by reviewing the current literature with particular focus on clinical studies published in qualified journals according to the impact factor. 

### 1.1. Upper GI Disorders

#### 1.1.1. Gastro-Esophageal Reflux Disease (GERD)

Gastro-esophageal reflux disease (GERD), is defined on the basis of chronic and recurrent typical symptoms, i.e., pyrosis and acid regurgitation as well as extra-esophageal manifestations, demonstrated to impair quality of life (QoL) [19].

The prevalence of GERD disease seems to show important geographical variations: when defined as pyrosis and/or acid regurgitation at least once a week, the prevalence in the Western countries predominantly ranges between 10% and 20% whereas in Asia the prevalence is quoted to be <5% [20]. In the past years, various longitudinal studies have identified different risk factors of GERD and, indeed, obesity is indicated as one of the significant risk factors in this disorder [6]. The raised prevalence of both GERD, in terms of symptomatic disease as well as complicated disease, and obesity has raised interestin the possible link between these two clinical scenario and in the possible explanations for this association. One of the main pathogenetic pathways linking obesity and GERD would seem to be the increased abdominal pressure leading to the relaxation of the lower esophageal sphincter (LES), and thus to the refluxing of the gastric content within the esophageal body [7]. In obesity, it is likely that the retrograde flow of gastric content into the esophagus is increased by an altered gastro-esophageal pressure gradients characteristic in these patients [21].

Therefore, studies conducted by high-resolution manometry have demonstrated that obesity was associated with occurrence of hiatus hernia (HH), a well-known risk factor for GERD. Indeed, it has been shown that body mass index and waist circumference are significantly correlated to the vertical separation of the high pressure zone of the LES and crural diaphragm, hypothesizing that this association was a result of the pressure stress due to the increased intra-gastric volume [22].

A recent study in which BMI, waist-hip-ratio (WHR), and GERD were compared, has pointed out that WHR is significantly associated with esophageal acid exposure and with impaired esophageal acid clearance concluding that WHR is a stronger predictor for pathological acid exposure at distal esophagus than BMI [23]. Despite anthropometric parameters, also body composition plays a pivotal role in the association between obesity and GERD. Actually, pathological parameters, in 24-hour pH impedance study, could be influenced by both mechanical and metabolic factors of visceral fat, which may affect patient selection for ambulatory reflux monitoring.

In this light, a very recent study has shown a significant association between abdominal fat and the site of mucosal breaks at esophago-gastric junction (EGJ) [24]. 

Moreover, when comparing the visceral fat with BMI and WHR as risk factors for GERD, evidence coming from a recent study has shown that the former is more important than BMI and waist-to-hip ratio as a risk factor for GERD [8]. Moreover, the abdominal obesity correlated with the severity of GERD based on Los Angeles classifications A, B, and C [25].

Abdominal obesity can therefore better justify the link between obesity and the complication of GERD such as Barrett Esophagus (BE) and esophageal adenocarcinoma. Indeed, it has been demonstrated, in GERD patients at high risk for BE, that the body fat is distributed more visceral than truncal as, in a recent study, waist circumference was demonstrated to be a significant risk factor for BE independently of BMI, while the association between BMI and BE vanished following the correction of the abdominal circumference [26]. Interestingly, visceral fat, in addition to mechanical pressure, seems to play a pivotal role as an active tissue from a metabolic point of view, and it has been clearly associated with the levels of adipo-cytokines in the serum including interleukin 6 and tumor necrosis factor α, the same mediators involved in GERD pathogenesis and consequent carcinogenesis [27].

It has been proven that weight loss is one of the few effective lifestyle modifications for GERD [28]. A recent study, carried out in patients with obesity and GERD, have explored the role of a weight control program in patients with reflux. The results of the study showed that GERD symptom severity, as well as body weight [29] improve in parallel with the improvement in lifestyle and with the consequent weight loss, thus confirming the pathogenetic link between obesity and GERD confirming the pathogenetic link between obesity and GERD.

#### 1.1.2. Functional Dyspepsia (FD)

Functional dyspepsia is a GI functional disorder characterized by symptoms, such as epigastric fullness and bloating, nausea, discomfort, and vomiting, which are provoked following food consumption [30]. The temporal correlation between symptoms and meal ingestion tends to drive physician in believing that functional dyspepsia was primarily caused by disorders of gastric motor functions [31]. Recent findings have demonstrated that the regulation of gastric functions, by means of vagal neurocircuits, is a plastic phenomenon, which can be modulated by several factors such as, peripheral and central input; in particular, vagal afferent sensitivity may be stimulated also by nutrient content [32]. Indeed, obesity has been identified as a risk factor for developing functional dyspepsia, particularly obesity is related to impaired gastric motility, gastric emptying rates, and to an increase in gastric volume [33]. In a retrospective study, aimed at comparing visceral and subcutaneous adipose tissue areas between patients complaining of functional dyspepsia, as diagnosed by the Rome criteria, and healthy subjects, it has been shown that visceral adiposity, but not subcutaneous adiposity, is significantly related with dyspepsia [34]. A very recent study confirmed the relationship between obesity and FD also in pediatric populations. In the above-mentioned study the prevalence of FD was assessed in children aged 4–18years oldsuffering from FD and obesity and in normal-weight children. The results showed no significant age and sex differences were found between the 2 groups; in contrast functional GI diseases were significantly more prevalent in obese compared to normal-weight children. A positive association was also found between obesity and irritable bowel syndrome, functional constipation but not for abdominal pain [35].

### 1.2. Lower GI Disorders

#### 1.2.1. Irritable Bowel Syndrome

Irritable bowel syndrome (IBS) is a GI disorder defined by chronic abdominal pain and changes in bowel habits without any known organic causes [36]. Among others etiopathogenetic factors, such as abnormal motility of intestine, inflammation, visceral hypersensitivity, neurotransmitter imbalance, and disturbance of gut-brain interactions, obesity seems to be involved in the pathogenesis of IBS. In the meantime, few studies explored the association between obesity and IBS. Data from literature have failed to show a possible association between IBS subtypes prevalence and BMI [37], however among IBS patients, those with abdominal obesity likely report frequent symptoms even after correction for possible confounders [38]. 

This finding confirms results coming from a population based-study showing a relationship between BMI and GI symptoms such as diarrhea [4]. Several factors are common in both obesity and IBS, such as a diet with high amount of refined carbohydrates, or lipids, and low in fiber, disorder of intestinal motility, gut microbiota alteration and inflammation which may explain the link between the two clinical conditions [38].

High serum low density lipoprotein (LDL) levels are an independent predictor of IBS in obese patients probably explained by altered fat absorption due to local low-grade inflammation in the gut or alteration of the gut microbiome [39]. Therefore, as blood lipoprotein levels are mostly regulated by the hepatocytes, non-alcoholic fatty liver disease, which modify the function of hepatocytes, is strongly related to obesity and it has been discussed in relation to IBS [40]. In an interesting study regarding the correlation between abdominal fat and IBS, visceral adiposity was more prevalent in patients with IBS symptoms in respect to the controls [41]. Alteration in visceral fat metabolism influences GI motility and stimulates production of adipokines and immunologic factors, thus emphasizing the role of obesity in the increased risk of IBS. In line with this finding, an increase in visceral adiposity was related to enhanced visceral perception of luminal stimula, dysmotility, and abdominal pain, which are frequent in patients with IBS [42]. Patients with IBS and related disorders have also increased bowel sensitivity to different stimuli; normally, intra-luminal lipids increase perception of concurrent intestinal stimuli and modulate intestinal motor reflexes, and these effects are amplified in IBS patients [43]. 

Interestingly, it seems that class II obesity is most common in post-infectious IBS (PI-IBS) than in non PI-IBS and controls having similar BMI, and the therapeutic response to IBS-therapy is less favorable in NPI-IBS and PI-IBS obese patients than in NPI-IBS and PI-IBS normal weight patients, supporting the possible correlation between these two pathological conditions [44]. Moreover, an interesting association between obesity and changes in microbiota composition has been hypothesized, confirming the possible link with IBS, in which intestinal microbiome plays an important role in generating symptoms. In germ-free mice, colonization with “obese gut microbiota” increased the fatty tissue and body weight, in contrast to colonization with “slim gut microbiota” [45]. Interestingly, “obese gut microbiota” is characterized by reduced number of Bacteroidetes and increased number of Firmicutes, which is associated with a greater capacity to extract energy from food, thanks to the increased degradation capacity of carbohydrates complexes [46]. Although data regarding IBS microbiota composition are still conflicting, also in these patients a reduced number of Bacteroidetes and an increased level of Firmicutes has been observed [47].

The role of altered microbiome in obese patients was confirmed by two animal models of insulin resistance, in which two weeks of treatments with norfloxacin and ampicillin had significantly improved fasting glycaemia and oral glucose tolerance, reducing liver triglycerides and plasma lipopolysaccharide (LPS) and increasing adiponectin also correlated with improved glucose tolerance [48]. Dysbiotic microbiota is also able to stimulate an increased intestinal mucosal penetration of different pathogens, and their metabolites, promoting a local low grade of inflammation, which is another interesting factor involved in this link between obesity and IBS, both characterized by the release of inflammatory mediators that can irritate intestinal nerve endings [49].

#### 1.2.2. Diverticulosis

Obesity is also associated to an increased risk of colonic diverticulosis [50]. Diverticulosis of the colon is an anatomic alteration of the colonic wall characterized by the presence of extroflexions that occur when colonic mucosa and sub-mucosa herniate through defects in the muscle layer of the colon wall [51]. In a prospective study aimed at assessing prevalence of diverticula in patients undergoing lower GI endoscopy it has been demonstrated that the prevalence of diverticulosis appeared to increase with a higher BMI compared with participants with a normal BMI. In particular, the multivariable adjusted odd ratios for diverticulosis were 3.02 (95% Confidence interval [CI], 1.33–6.88) and 4.43 (95% CI, 1.88–10.49) among participants with a BMI of 25.0–29.9 kg/m^2^ and 30.0 kg/m^2^ or greater, respectively [9]. Different plausible mechanisms linking obesity and diverticulosis have been proposed. Difference of gut bacterial flora between obese and non-obese subjects may play a role in the development of colonic diverticulosis. Previous studies have demonstrated a higher concentration of methane in the colon of obese subjects with a positive correlation with BMI, which is believed to be a result of altered gut bacterial flora. The higher concentration of methane may increase intraluminal pressure and ultimately contribute to the development of colonic diverticulosis [52]. Moreover, the excess of visceral fat in abdomen might increase the intra-abdominal pressure in obese compared with subjects with normal BMI. The increased intra-abdominal pressure can lead to increased intraluminal pressure and, thus, the higher likelihood of diverticula formation [53]. Finally, it has to born in mind that obesity and diverticulosis share common predisposing factors such as poor eating habit (including inadequate fiber consumption) and physical inactivity. In addition, to the higher risk of occurrence of diverticulosis in obese patients, it has been demonstrated the obesity might also be associated with a higher risk of complication. Indeed, an association has been demonstrated between an increased incidence and severity of complicated diverticular disease [54]. In particular data coming from a population study have shown that among several factors such as hypertension, dyslipidemia, and chronic kidney disease, increasing BMI and increasing visceral adipose tissue were associated with diverticular bleeding [55]. 

#### 1.2.3. Inflammatory Bowel Disease (IBD) 

The incidence of IBD is increasing in parallel with overweight and obesity, with approximately a percentage of 15–40% overweight in patients with IBD [56] The global increasing incidence in IBD seems to be associated with western lifestyle; in particular, it is well known that diet can shape the microbiota composition and impact on host-microbe interactions [57]. It is well recognized that specific dietary factors such as protein and fat can result in increased production of bacterial metabolites, that may be harmful to the gut, stimulating inflammatory processes. On the other hand, bacterial fermentation of non-digestible carbohydrates results in short chain fatty acids (SCFAs), which are an energy source for host epithelial cells and act as signaling molecules with anti-inflammatory, immunomodulatory, anti-oxidative, and improved mucosal barrier effects. Moreover, fat can have effects on the microbiome by release and conversion of bile salts thus negatively modulated the microbiota composition [14].

Results coming from a recent study have pointed out that obesity is a risk factor for occurrence of IBD, mainly Crohn’s disease in respect to Ulcerative colitis [58]. Visceral adiposity is the metabolically active fraction, and could be more predictive of the risk of developing IBD that general obesity determined by the BMI. Indeed, there is evidence that obesity is able to influence not only the occurrence but also the progression of IBD; it has been shown that visceral obesity, in patients with CD, is associated with a higher probability of surgery and of penetrating disease and in ulcerative colitis with an increased risk of relapse [59]. However, in a meta-analysis regarding the evolution of IBD disease, the results showed that obese patients undergone surgery less frequently than non-obese patients (RR 0.82; 95% CI 0.72–0.93). This could be explained by considering that a lower BMI could be the result of the inflammatory progression and that the obesity is a reflection of a less serious IBD [60].

Moreover, obesity might also impair clinical response to treatment, indeed in a longitudinal study in IBD patients, obesity was not only related to higher clinical activity at baseline evaluation, assessed by using validated disease activity indexes, but also to a higher risk of relapse and remaining persistently active compared with patients with normal BMI at 12 months follow-up [61]. These findings confirm previous data showing that obesity can negatively affect response to biologic therapy in patients with ulcerative colitis [62]. Moreover, specific to surgical patients, multiple lines of evidence suggest that obesity may negatively influence surgical outcomes, specifically when obesity is defined according to volumetric analysis of fat distribution, rather than solely BMI [63]. 

### 1.3. Pancreatitis

Obesity is recognized as a persistent state of chronic low-grade inflammation, through a systemic and paracrine increase of cytokines, chemokines, and adipokines. Obesity increases leptin secretion from adipocytes and proinflammatory cytokines, such as tumor necrosis factor and interleukins 1 and 6, from macrophages and leukocytes [15]. Obesity, besides its direct impact on inflammation, is also able to modulate the pharmacokinetics of biologic agents, resulting in rapid drug clearance [64]. Thus, obesity could negatively affect both inflammatory GI disease and response to medical therapy.

Intra-abdominal white adipose tissue (WAT) is not considered to be an isolated tissue any more, which store fatty acids serving as passive energy reservoir. The new understanding of WAT as an active secretory organ have changed the point of view also on mesenteric adipocytes no longer considered as simple passive cells in GI disease. There is evidence that several adipose tissue-derived proteins are involved in multiple metabolic and inflammatory pathways [65].

For example, the growing pandemic of obesity has increased acute pancreatitis incidence and severity. The increased incidence is due to increased risk of gallstones, hypertriglyceridemia, diabetes, medications, and bariatric surgery for weight loss interventions. Moreover, obesity worsens acute pancreatitis severity by allowing unregulated lipolysis of visceral fat enriched in unsaturated triglyceride; these alterations are responsible for the occurrence of necrosis [16].

### 1.4. Nonalcoholic Fatty Liver Disease

Liver function might be impaired by excess body weight, in fact it has been demonstrated that overweight and obesity are associated with elevated biochemical markers of liver damage both in adults and in adolescent [66,67]. In particular, consistent data from literature have pointed out a clear relationship between obesity and nonalcoholic fatty liver disease (NAFLD) being insulin resistant is a main driven factor [68]. The clinical relevance of the nonalcoholic fatty liver disease (NAFLD) derives mostly from its high prevalence in the general population, its associated risk for progression to nonalcoholic steatohepatitis (NASH), hepatic fibrosis, and cirrhosis [69]. Moreover, the rising incidence and prevalence of childhood obesity suggests that NAFLD is likely to become an even greater contributor to society’s burden of liver disease in the future. Intestinal microbiota, beside the main function of metabolize dietary fiber, is also able to collect energy from dietary sources, indeed it has been demonstrated that obesity may modulate microbiota leading to accumulation of triglycerides in the hepatocyte. Moreover, the modification of the activity of the microbiota in obese patients is related to the impairment of intestinal permeability. Reduced intestinal barrier function leads to hepatic exposure to gut-derived products, which stimulates liver cells to generate inflammatory mediators that inhibit insulin actions [70]. In obesity, the insulin sensitivity of peripheral tissue is impaired because of the adipokines, soluble factors released form adipose depots [71]. Insulin resistant promotes hyperglycemia thus leading the pancreas to produce more insulin to maintain glucose homeostasis. However, hyperinsulinemia also promotes lipid uptake, fat synthesis and fat storage. Recently, it has been carried out a study in mice, aimed to assess the role of long-term (80 weeks) high fat diet (60% fat, 20% protein, 20% carbohydrate) on liver pathology, fibrosis, inflammation, and endoplasmic reticulum (ER) stress. A microbiome analysis has also been performed. The results showed that high fat diet (HFD) feeding promotes obesity, insulin resistance, ER stress, alterations in gut bacterial composition, and NAFLD. In particular, histopathological assessment demonstrated that liver of old mice fed with HFD showed extensive steatosis, portal and lobular inflammation with cell injury (ballooning degeneration of the hepatocytes), and evident fibrosis [72]. 

Pathogenic role of obesity in NAFLD has been confirmed by outcome study focused on the role of lifestyle changes and dietary modification the treatment of this condition [73]. Indeed, both diet and exercise are recognized as first line therapy; in adults with NAFLD, exercise regimens that improve fitness may be sufficient to reduce hepatic steatosis [74,75]. Indeed, it has been widely demonstrated that, the beneficial effects of the Mediterranean diet on NAFLD could be explained by both the promotion of weight loss and the provision of nutrients and bioactive compounds. Indeed, it has been demonstrated that polyphenols, vitamins, and terpenes, may display an anti-inflammatory effect in the liver thus leading to a reduction in the oxidative stress [76].

### 1.5. GI Cancer

Cancer is the major causes of death worldwide. Moreover, the number of cancer is increasing because of the growth of the populations, of age, and the adoption of lifestyle behaviors linked to increased cancer risk. A causal association between body fatness and different types of cancer is supported by strong evidence; moreover, excess weight is also well-known as a risk factor for cancer mortality overall [10]. As far as concerns GI cancer, being overweight and obesity have been associated with an increased risk of developing esophageal, gastric, hepatocellular, pancreatic, and colorectal cancer. Based on available data, it is reasonable that strategies aimed at encouraging the consumption of a healthy diet, such as Mediterranean diet, and the practice of regular physical activity are needed for weight maintenance and possibly weight loss in obese patients.

Indeed, to date, it is well recognized that avoiding excess body fatness is a way to reduce risk of cancers occurrence together with other lifestyle items such as avoiding smoking and alcohol abuse. An interesting recent investigation has also pointed out that overweight in youth and young adulthood is related to increased risk of many cancers linked to adult weight, this evidence supports the need of preventing strategies [77]. Indeed, it has been demonstrated that being physically active since childhood, may lower the risks of developing breast, colon, and endometrial cancer [78].

One plausible molecular mechanism for obesity-associated carcinogenesis is the chronic inflammatory state, which is the result of the activity of the visceral adipose tissue leading to the release of inflammatory cytokines and mediators [79]. Moreover, insulin resistance plays a promoting role in the pathogenesis of GI cancers [80]. 

Colon cancer is the third most common cancer among men and women and the second most common cause of cancer death [81]. Consistent data from the literature have pointed out a dose-response relationship between BMI, as well as waist circumference, and increased risk of the disease, with risk generally higher in men than in women [82]. A meta-analysis of 30 prospective studies by Larsson and Wolk reported that for every 5 kg/m^2^ increase in BMI, the risk of colorectal cancer increased by 30% [11]. Among several factors, a very recent population based study carried out in the USA confirmed that obesity, diabetes mellitus, family history of other malignancy, and smoking independently increase the odds ratio for the occurrence of colorectal cancer [83]. Inflammatory cytokines appear to play a key role in linking obesity and colorectal carcinogenesis particularly so for IL-6 and tumor necrosis factor-α [84,85]; a recent study also describing IL-13 as a potential factor involved in the development of obesity-related colon cancer onset [86]. As it was the case for colon rectal cancer, also for the gastro-esophageal cancer a positive relationship between abdominal obesity and cancer occurrence has been described. Visceral fat may impair the gastro-esophageal junction not only mechanically but also systemically via metabolic/inflammatory pathways involving free fatty acids, tumor necrosis factor α (TNFα), leptin, and resist in, and insulin-like growth factor (IGF-1) [87]. Overweight and obesity is also associated with higher risk of liver cancer as well as to negative clinical outcomes, such as time to recurrence, disease-free survival, and overall survival among patients with hepatocellular carcinoma and pancreas cancer [88,89].

### 1.6. Role of Lipid and Dietary Pattern in GI Symptoms

**Figure 3:**
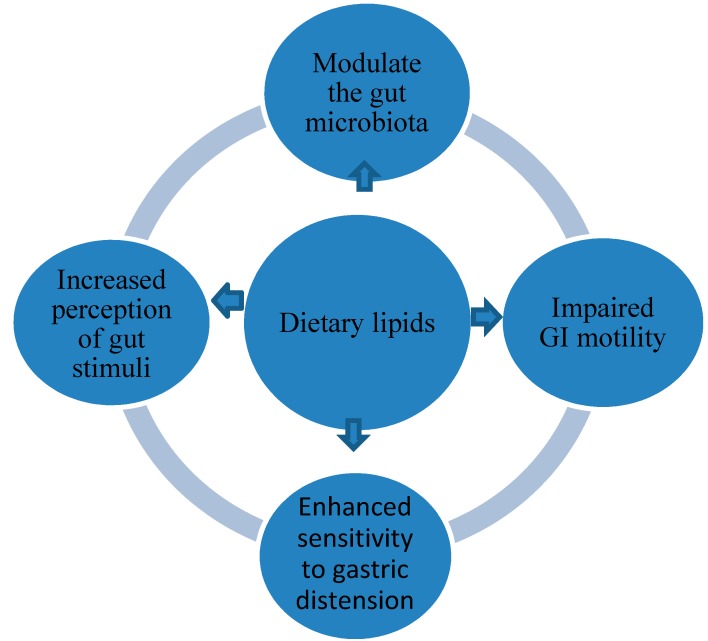
Mechanisms linking dietary lipids and GI symptoms.

Obese people often have increased habitual energy and fat intakes, particularly obese patients show a dietary model characterized of lower intake of fruit and vegetable and high intake of foods rich in carbohydrates and fat [12]. It has been shown that foods with a high content of fat can stir up GI symptoms in particular functional GI disorders as shown in Figure 3 [90]. 

Indeed, in patients with IBS, it has been demonstrated that intestinal lipids raise intestinal perception and sensitivity of gut stimuli, by their ability to sensitization of gut mechanoreceptors [91]. Fat can also affect the quality of perception of upper gut sensations. Such as, it has been demonstrated in patients with functional dyspepsia compared to healthy volunteers, that infusion of lipid within the duodenum incites strongly greater symptoms, such as nausea, epigastric fullness and bloating. Moreover, fat may also have enhanced sensitivity to gastric distension [92]. In addition, both nausea and pain scores were lower following consumption of a high-carbohydrate yogurt, with the same volume and the same caloric to the high-fat yogurt. Lipids are also able to enhance perception of intestinal stimuli as well as modulate intestinal motor reflexes in patients with IBS [93]. Duodenal lipid increases the frequency of transient lower esophageal sphincter relaxations in patients with GERD, associated with an enhancement number of reflux episodes [94]. 

Lipid may also be able to affect GI motility, in particular, lipids can impede small bowel motility, in fact intestinal gas transit is slower by fat leading to bloating. On the other hand, duodenal lipids boost the movement in the colon [95]. It has been hypothesized that the gut hormones associated to lipid presence such as, GLP-1 and PYY might be the link between fat an occurrence and severity of symptoms in functional GI disorders [17].

Nutrition and high intake of saturated fats, besides the important environmental factors for obesity and for the development of GI symptoms, might also be able to modulate the gut microbiota. Recent data coming from experimental studies in mice have pointed out the role of dietary lipids in altering gut composition and functions. In particular, it has been shown that the microbiota composition both in the stomach and in the gut was clearly different between long- term high fat diet and short-term high fat diet which was related to the deterioration of metabolic profile [13]. This data is in keeping with previous findings showing that a 5-week high fat feeding significantly altered the body weight and metabolic parameters in mice. Interesting the subsequent introduction of a low fat diet for 2 weeks normalized body weight gain to that of the control-diet fed mice as well as restored the metabolic parameters to the levels of the control group [96].

## 2. Conclusions

A consistent body of evidence supports the relationship between excess adiposity and elevated risks of developing gastrointestinal disease. The presence of obesity not only increases the risk of developing gastrointestinal diseases but also is also associated with more severe disease phenotypes and a lower response to treatments, thus leading to more unfavorable clinical outcomes in general with consequent clinical and economic burden. Additionally, the relationship between obesity and GI disease is also true for the pediatric population. These results support the urgent need to implement not only effective therapeutic strategies but also prevention programs for childhood obesity.

## Figures and Tables

**Table 1:** Categories of overweight and obesity according to body mass index (BMI).

Overweight: 25.0–29.9 kg/m^2^
Class 1 obesity: 30.0–34.9 kg/m^2^
Class 2 obesity: 35.0–39.9 kg/m^2^
Class 3 obesity ≥40 kg/m^2^

**Table 2:** Some of the mechanisms linking obesity and GI disease.

Factor	Mechanism	GI Disease	References
**Mechanical**	Increase abdominal pressure Lead to the relaxation of the lower esophageal sphincter (LES) Increase the risk of occurrence of hiatus hernia	GERD Diverticular disease	Emerenziani S. et al., 2013 [6] Pandolfino JE et al., 2006 [7] Ze EY et al., 2017 [8] Mashayekhi R. et al., 2018 [9]
**Pro tumoral**	Visceral fat releases pro-tumoral factors	GI cancer	Lauby-Secretan B. et al., 2016 [10] Larsson SC et al., 2007 [11]
**Dietary factors**	Increased perception of concurrent intestinal stimuli Modulation of intestinal motor reflexes Inhibition of small bowel motility and delay of intestinal gas transit. Enhanced gastro-colic reflex Modulation of microbiota composition	IBS Functional Dyspepsia GERD	Stewart J.E., et al., 2011 [12] Cong H et al., 2018 [13]
**Low-grade inflammation**	Visceral fat release of pro-inflammatory cytokines such as tumor necrosis factor and interleukins 1 and 6	IBD Pancreatitis NAFLD	Staley C, et al., 2017 [14] Kredel L. et al., 2014 [15] Khatua B. et al., 2017 [16]
**Adipocytes-released peptides**	Control of GI motility	GI motor disorders	Feinle-Bisset C. et al., 2016 [17]

GERD: Gastroesophageal reflux disease, IBS: Irritable bowel syndrome, IBD: Inflammatory bowel disease, GI: Gastrointestinal, NAFLD: Nonalcoholic fatty liver disease.
